# Miniaturized Hyperspectral Imager Utilizing a Reconfigurable Filter Array for Both High Spatial and Spectral Resolutions

**DOI:** 10.1021/acs.nanolett.4c01075

**Published:** 2024-08-30

**Authors:** Tingbiao Guo, Zijian Lin, Zhi Zhang, Xiao Chen, Yuan Zhang, Zhipeng Hu, Ruili Zhang, Sailing He

**Affiliations:** † Centre for Optical and Electromagnetic Research, College of Optical Science and Engineering, National Engineering Research Center for Optical Instruments, Zhejiang University, Hangzhou, 310058, China; ‡ Taizhou Institute of Medicine, Health and New Drug Clinical Research, Taizhou Hospital, Zhejiang University, Taizhou, 318000, People’s Republic of China; § National Engineering Research Center for Optical Instruments, Zhejiang University, Hangzhou, 310058, China; ∥ Department of Electromagnetic Engineering, School of Electrical Engineering, KTH Royal Institute of Technology, Stockholm, SE-100 44, Sweden

**Keywords:** hyperspectral imaging, array filter chip, vanadium oxide, reconfigurable filter

## Abstract

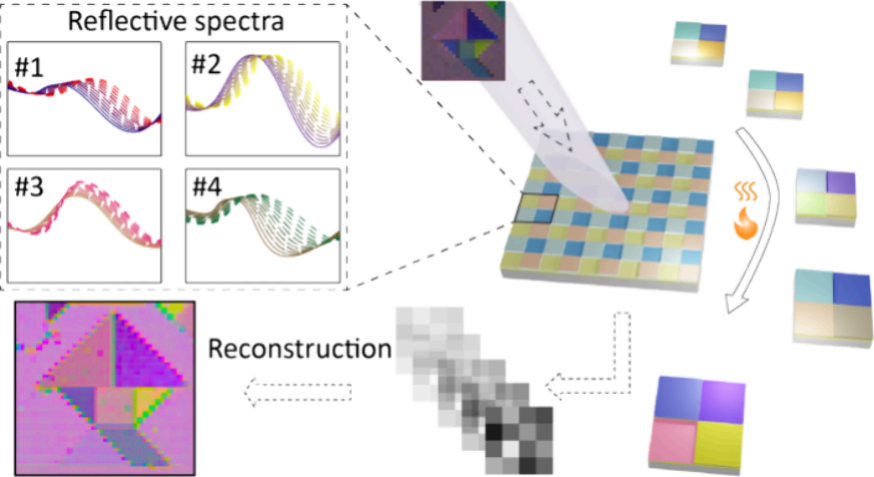

Miniaturized hyperspectral imaging based on filter arrays has attracted much attention in consumer applications, such as food safety and biomedical applications. In this Letter, we demonstrate a miniaturized hyperspectral imager using a reconfigurable filter array to tackle the existing trade-off issue between the spectral and spatial resolutions. Utilizing tens of intermediate states of a vanadium dioxide cavity, we increase the total number of physical spectral channels by tens of times from a 2 × 2 mosaic filter unit, providing both high spatial and spectral resolutions for spectral imaging. The reconfigurable filter has a good spectral resolvability of 10 nm in the visible range with a wavelength inaccuracy of less than 2.1 nm. Hyperspectral imaging is demonstrated with a frame rate of 4.5 Hz.

Hyperspectral imaging is a booming technique with which the spatial and spectral information on the objects can be obtained simultaneously, enriching traditional imaging technology and providing detailed features for applications such as precision agriculture,^1^ food safety and inspection,^2^ environmental monitoring,^3^ and biomedical imaging.^4,5^ Conventional technology (e.g., push-broom method) mainly uses a grating or prism for spectrum capturing and a mechanical scanning component for image acquisition.^6^ In this configuration, spectral resolution is mainly determined by the quality of a grating and the length of the optical path (Figure 1a). To achieve a hyperfine spectral resolution, small slit and premium gratings are indispensable, leading to high cost, poor stability, bulky volume, and importantly, low signal-to-noise ratios (SNR).

**Figure 1:**
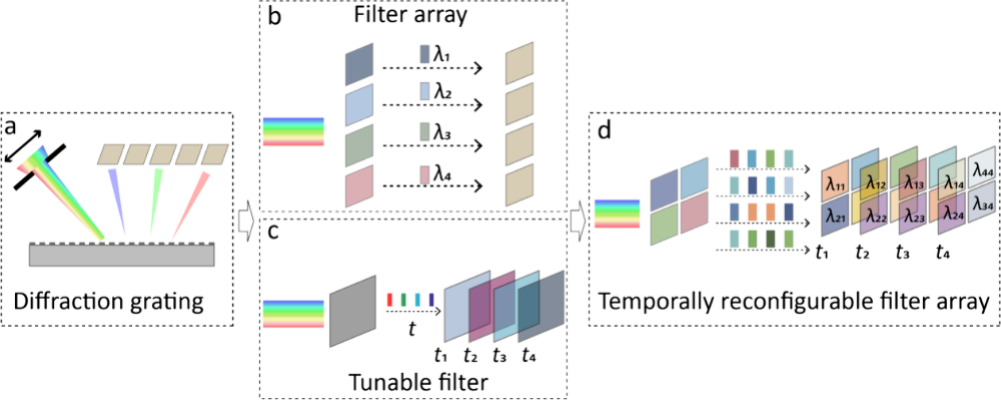
Principle of the hyperspectral imaging system based on different approaches: (a) Diffraction gratings; (b) Filter arrays; (c) Tunable filters; (d) Temporally reconfigurable filter array.

Over the past decade, computational spectral reconstruction has become an intriguing technique along with burgeoning miniaturized spectrometer research. Filter arrays including multilayer thin film filters,^7,8^ photonic crystal cavities,^9^ metasurface filters,^10,11^ and even nanowires^12,13^ and quantum-dot arrays^14^ have been demonstrated and shown unequivocal superiority considering the low SWaP (size, weight, and power) (Figure 1b). Nevertheless, these snapshot realizations come at the expense of spatial resolution. To obtain a higher spectral resolution, massive filters (detector pixels) are required, resulting in lower spatial resolution caused by pixel merging. This trade-off hinders snapshot filters for practical applications where spatial resolution plays a vital role, such as small target detection and pathological examination. Moreover, monolithically integrating numerous filters on a single chip poses fabrication challenges and complexities.

As an alternative strategy, hyperspectral imaging can also be achieved by using active filters with reconfigurable responses (Figure 1c). By controlling the external stimulus, unknown spectral components are temporally separated and detected with high spatial resolution. However, to reconstruct the input signal, multiple intermediate filtering states with low correlation coefficients are prerequisites for the tunable filters, which impose rigorous requirements on the active material. Due to this limitation, the choice of materials for a tunable filter is limited to a few categories like liquid crystals,^15−17^ acoustic-optical materials,^18^ and more recently the emerging 2D materials^19,20^ and perovskite quantum dots.^21^ In addition, for tunable filters with a fixed geometry, the tuning range is usually restricted to a narrow band, further limiting their use in broadband scenarios.

In this Letter, by introducing the active material into the filter array and combining spatial and spectral multiplexing features, we demonstrate a miniaturized hyperspectral imager utilizing a temporally reconfigurable filter array (TRFA) composed of VO_2_ to increase the physical spectral channels (Figure 1d). In this configuration, four VO_2_-based filters as a unit are arranged as a mosaic type. By triggering an insulator to metal transition,^22^ extensive intermediate filtering states can be achieved for each VO_2_ filter. In this way, aiding with the multiplex of both spatial and temporal modulation, hundreds of effective wavelength channels can be achieved in a four-filter unit cell. This hybrid modulation greatly increases the spatial resolution compared to the conventional filter array approach, enriching the toolbox for diverse spectral imaging scenarios. It is noted that this is an extension (e.g. earlier one-dimensional spectral detection is extended to the reconstruction of the hyperspectral cube for hyperspectral imaging) with a detailed description of the spectral tuning part of our earlier work.^29^ The aim of this paper is hyperspectral imaging (rather than the row–column addressable electronic paper/color display). Therefore, here we use only a single large heater of millimeter size (instead of the microheater array) to trigger the whole filter array. Furthermore, the phase transition is induced by a single voltage (instead of many different voltages), and by capturing 41 distinct intermediate states during the heating process (rather than waiting for a stable state). As an experimental demonstration for hyperspectral imaging, a color object was reconstructed with high color fidelity with a sampling speed of 220 ms. The spectral feature of 10 nm can be distinguished within a dynamic range of 300 nm. Combining the ease of large-scale manufacturing, such a miniatured hyperspectral imaging method shows a powerful potential for medical diagnostics (e.g., endoscopy images for rectal cancer), microscopy, precision machining, and gas detection.

Figure 2 shows the schematic of the present design. For each unit cell, four basic filters with various VO_2_ thicknesses are monolithically arranged in a mosaic pattern on a silicon oxide substrate. The basic filter consists of a lossy cavity with a VO_2_ layer on a thick silver (Ag) film (Figure 2a). Due to the large refractive index change along with the phase transition of the VO_2_ materials (Supporting Information, Figure S1), the cavity possesses a significant change in reflection and colors.^23,24^Figure 2b shows the fabricated TRFA chip and the corresponding optical images before and after the phase transition.

**Figure 2:**
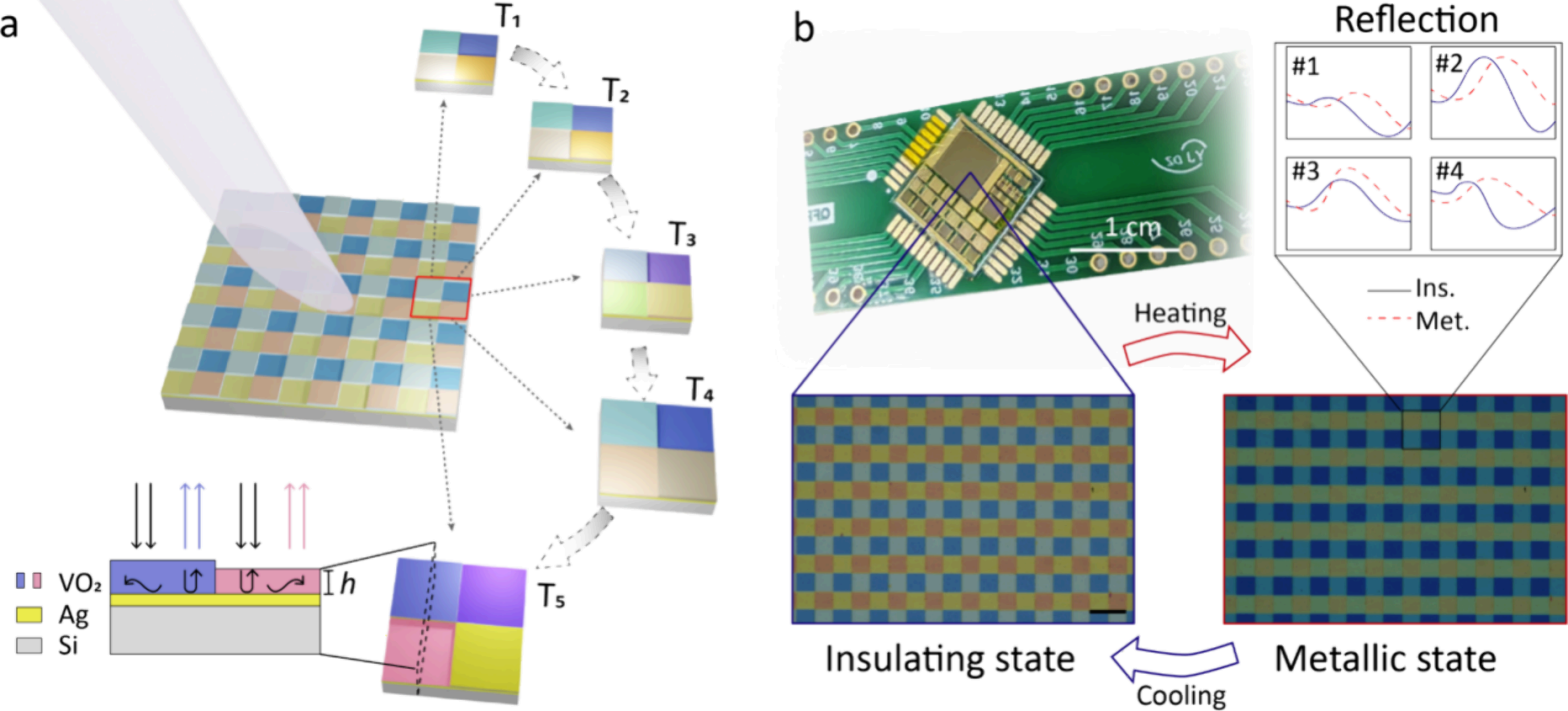
(a) Schematic of the temporally reconfigurable filter array. (b) The optical images of the fabricated TRFA chip. Scale bar: 20 μm in (b).

Such a hybrid approach has the advantage of higher spatial resolution and broadband working range for the hyperspectral imager compared to the separate method of filter array or tunable filter. By precisely controlling the temperature, one can obtain multiple intermediate states between the insulating state and metallic state of the VO_2_ material, which correspondingly expands the spectral channel for each filter. For the optical spectrum of incident signal *I*(λ) to be measured, the reconstruction principle is represented below:^9^
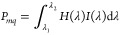
1where *P*_*mq*_ is the detected power of the detector after the *m*th color filter when the VO_2_ filter is at the *q*th intermediate state, *H*(λ) is the response function of the system, and λ_1_ and λ_2_ are the lower and upper limits of the working range. The above equation can be discretized as^9^
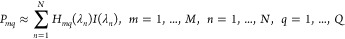
2where *H*_*mq*_(λ_*n*_) = *F*_*mq*_(λ_*n*_)*R*(λ_*n*_)*L*(λ_*n*_), and *F*_*mq*_(λ_*n*_) is the reflective spectrum of the *m*th color filter at *q*th filtering state at λ_*n*_. *R*(λ_*n*_) is the response of the detector and *L*(λ_*n*_) is the optical response of the system at λ_*n*_. *M* defines the total number of color filters in a unit; here, *M* = 4. *Q* is the total intermediate state number of a VO_2_ filter; in this paper, we set *Q* = 41. The equivalent filter channels are *M* × *Q* = 164. *N* is the discrete wavelength channel that needs to be resolved. The original spectrum can be retrieved accurately by solving these equations, and the resolution is related to the correlation coefficient.

Figure 3a shows the temperature-dependent reflectance spectra and corresponding color of four filters under different temperatures ranging from 30 to 75 °C. The thicknesses of the VO_2_ layer for the four filters are 50, 75, 100, and 125 nm, labeled as S1–S4, respectively, and the width of a unit array filter is 20 μm. Due to the hysteresis effect of VO_2_, the filters show different spectral responses in the heating and cooling process and possess different spectral responses (see Supporting Information, Section 2). Yet, the hysteresis effect does not expand the tuning range; we only choose intermediate states during the heating process for spectral reconstruction in the next section. When the temperature increases, the color appearances become darker for all filters, along with a resonance shift, due to the large loss for the metallic state (Figure 3a–d). Although the color change range is not large, there exists excellent spectral modulation at certain specific wavelengths, such as S1 at 600 nm and S2 at 650 nm, with the modulation |*R*_h_ – *R*_l_|/*R*_l_ as large as 1000%, where *R*_h_ and *R*_l_ mean the reflective spectra at high and low temperatures (Figure 3e). This also makes it suitable for an intensity modulator in the visible region. The corresponding color change trajectories are also labeled in the CIE 1931 color space, as shown in Figure 3f. Although the color gamut of the present device is somewhat narrow, it could be enlarged with a multilayer design for color display applications (Supporting Information, Section 3).

**Figure 3:**
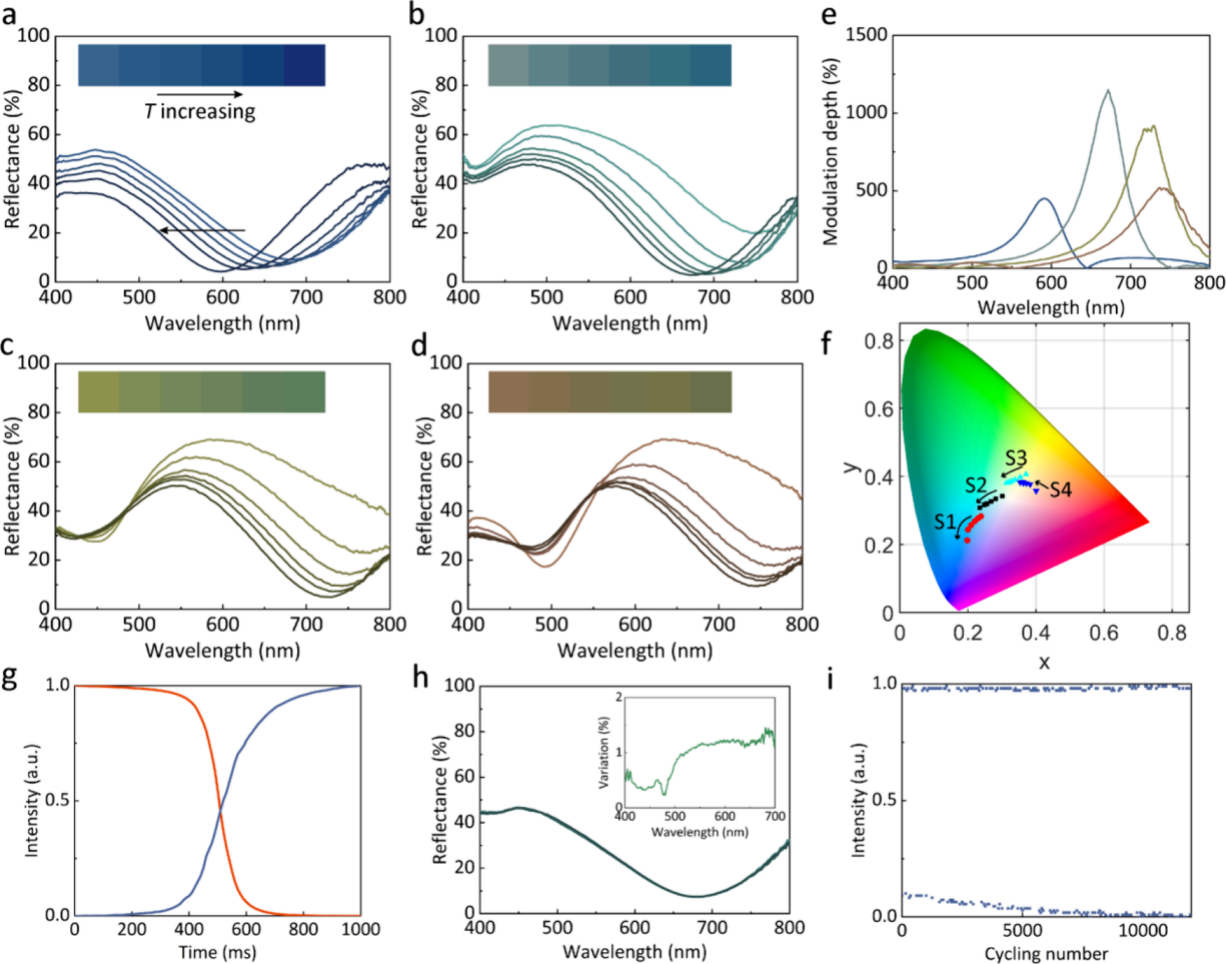
(a–d) The reflectance of the fabricated filters as the temperature increases, as well as the optical images as inset. (e) The modulation depth for different filters. (f) The color change trace in the CIE 1931 diagram for the four filters. (g) The response time of the filters. (h) The reflective spectra of the filter at a chosen temperature measured for ten cycles.The inset shows the variation of the reflective spectra. (i) The durability of the VO_2_ filters.

Instead of sampling the reflective response at different temperatures one by one, we used a transient sampling scheme. For this, an indium tin oxide (ITO) heater is fabricated beneath the filters and wire-bonded, as shown in Figure 2b. A pulsed signal (*U* = 4 V) was applied on the ITO heater, and we captured the transient reflective response during the heating process. The response time of our device is around 230 ms (Figure 3g). It is defined as the average rising and falling time measured from 10% (90%) to 90% (10%) of the intensity. The response time of the present filter is limited only by the response time of the heater. A smaller microheater and more delicate thermal management could be further utilized. The switching speed of a microheater is explored in Supporting Information, Section 4. It shows a response time of up to several kilohertz, where we can apply different signals to obtain each intermediate state one by one with various input voltages. As the resistance of our heater is around 60 Ω, the power consumption is 78 mJ for a single hyperspectral shot. The repeatability of the spectral response is quite critical for reconstruction. To explore this, we measured the spectra of our filters at chosen temperatures for ten cycles in situ. The variation of the spectra is smaller than 2%, indicating good reversibility and reproducibility of our VO_2_ filters (Figure 3h). The reproducibility of the dynamic response during the heating process was also verified, ensuring the consistency of the calibration and measurement process. All of these details could be found in Supporting Information, Section 5. The durability of the VO_2_ filter is also evaluated experimentally. We subject the VO_2_ filter to over 12000 heating–cooling cycles with a microheater. As shown in Figure 3i, no apparent performance degradation was found for the present device. Angle sensitivity is indeed a concern for an optical filter, and the simulation and experimental results for the angle-dependent optical response of our filter can be found in Supporting Information, Section 6. No evident wavelength shift occurs for our filters within an incident angle of 22°. As we used an objective with the numerical aperture (NA) of 0.3 (∼17.5°) for all experiments, the angle-dependent effect can be ignored.

To validate the function of TRFA for spectrum recovery, we built a prototype setup (Figure 4a), and different narrowband and broadband signals were used as the input signals for characterization. Due to the different responses of the filters, the black and white (B/W) camera would capture different intensity patterns for different input signals. By applying a voltage onto the heater to trigger the phase transition, these patterns would vary accordingly (Figure 4b). The reconstructive spectra for both the broadband and narrowband optical signals are shown in Figure 4c,d (details about calibration and reconstruction can be found in Supporting Information, Section 7). As a comparison, the original spectra calibrated by a commercial spectrometer are also depicted (dashed lines). The root-mean-square error (RMSE) of the measured (dashed) and ground truth spectra ranges from 0.011 to 0.067, with an average of 0.028. The largest peak inaccuracy is around 2.1 nm. The broadband spectra have also been well reconstructed and consistent with the reference spectra although with a larger average RMSE of 0.123 (Figure 4d). The reconstruction performance for broadband signals is worse than that for narrowband signals. This originates from the larger inaccuracy during the measurement of the broadband signals. A more detailed discussion can be found in Supporting Information, Section 8. The theoretical resolution of our system can be estimated by the equation below:
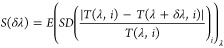
3where *T*(λ,*i*) is the reflection coefficient at wavelength λ under filtering channel *i*. *E*(•)_λ_ represents the average over wavelengths, and *SD*(•)_*i*_ means the standard deviation over filtering channels *i* (filter number *M* multiplies intermediate states *Q*). The spectral resolution can be estimated by δλ at which *S*(δλ) is equal to the noise ratio of the detector. The detector we used in the experiment has a noise around 1%.^17^ As shown in Figure 4e, the estimated spectral resolution of the device could be ∼2 nm. To obtain a better reconstruction performance, the supercell units can be designed by optimization methods (Supporting Information, Section 9).

**Figure 4:**
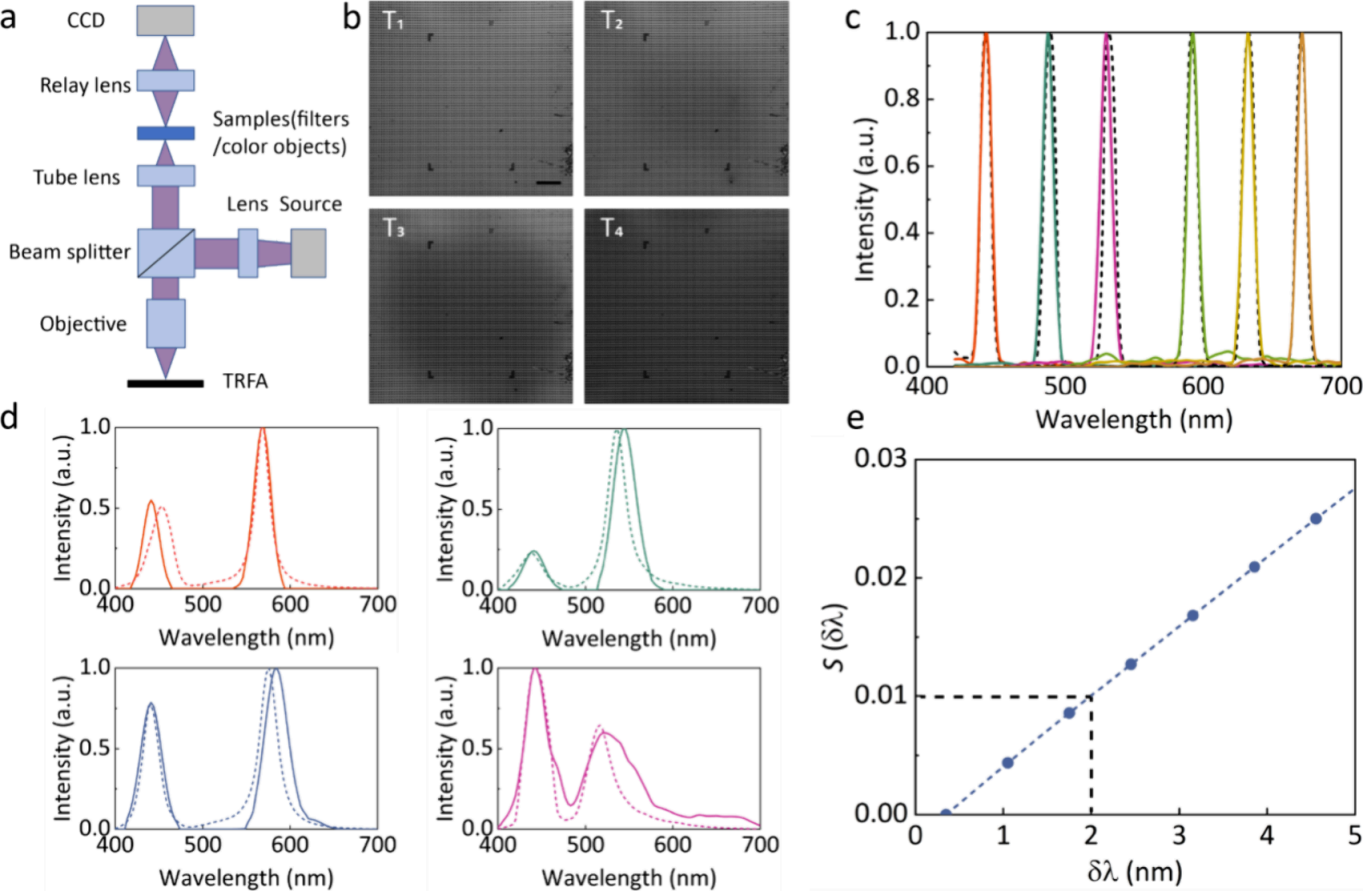
(a) Setup for spectrum reconstruction. (b) Original B/W pictures for filters at different temperatures with an illumination wavelength of 600 nm. (c, d) Reference (dashed lines) and recovered (solid lines) spectra for both the narrowband and broadband input signals. (e) Calculated *S*(δλ) used to estimate the spectral resolution of our system. Scale bar: 200 μm in (b).

Then we demonstrated hyperspectral imaging. A colorful object made of Fabry–Perot cavities was used as a sample and laid at the intermediate image plane after a relay lens. Figure 5a shows the original image (i), the B/W image captured by our system (ii), and the reconstructed false-color image (iii). Rich and uniform colors can be recovered. The mosaic in the image is due to the large magnification of the setup. To simplify the image processing, a filter unit is magnified to 200 μm (by width), occupying 40 pixels on the sensor in the experiment; thus, the spatial resolution for spectral imaging is largely decreased. For future work, a high-quality transmissive temporally reconfigurable filter array fabricated pixel-wise on an image sensor may also be a choice. More discussion can be found in Supporting Information, Section 10. In this manner, the theoretical sensing pixels occupied by a TRFA unit are four. To verify the spectral reconstruction of different points in the image, we select two points and show the corresponding spectra in Figure 5b. Good consistency between the reconstructed and reference spectra demonstrates the validation of our tunable filter array for spectral imaging.

**Figure 5:**
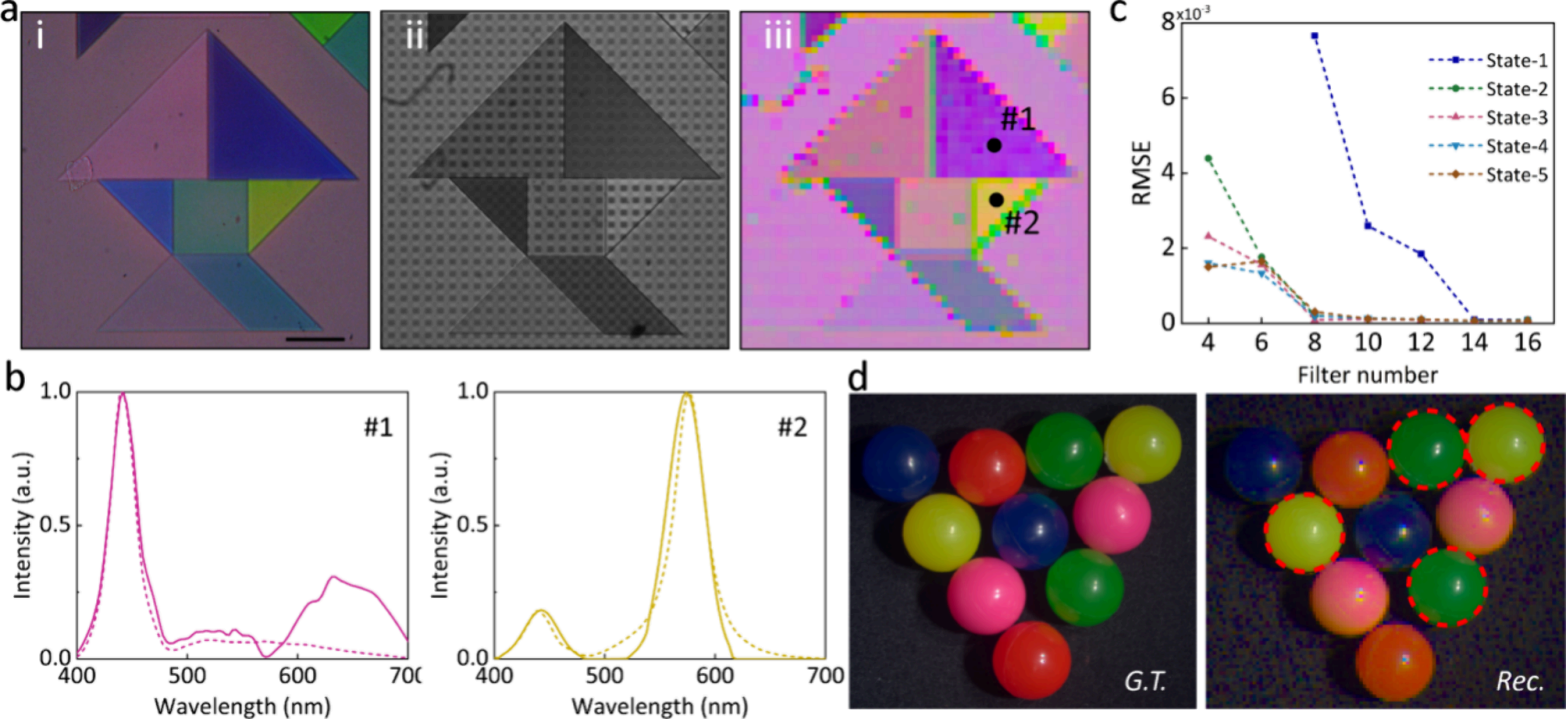
(a) Original image (i), the captured B/W image (ii), and the pseudocolor image recovered by our system (iii). (b) Reconstructed spectra (solid lines) and reference spectra measured by a commercial spectrometer (dashed lines) at points 1 and 2 marked in (iii). (c) Reconstruction performance for various combinations of filter numbers and the intermediate states. Different curves show the intermediate states from 1 to 5. (d) Simulated image results obtained by different reconstruction strategies for different regions. The dashed circles are the regions of interest reconstructed with 4 filters and 4 intermediate states. Other regions are reconstructed by 16 filters with 1 state. Scale bar: 100 μm in (a).

With the existence of multiple intermediate states, the spectrum recovery algorithm for TRFA can be utilized more adaptively. To further demonstrate this scalability of TRFA, more numerical simulations are performed. In the new configuration, 16 different filters with minimal correlation coefficients are chosen as the unit cell by particle swarm optimization (see the Supporting Information, Section 11). By delicately combining the filter number and intermediate state number in the spectral imaging algorithm, print-on-demand spectral, spatial, and temporal resolution can be obtained adaptively. The simulated RMSEs for various combinations of different filters and intermediate states are shown in Figure 5c. As expected, either a large number of states or a large number of filters are beneficial for better reconstruction. Figure 5d shows the simulated image results obtained by this adaptive method. Assuming that one would like to observe the details of the green balls (the region of interest), the balls can be reconstructed by a tunable mode (4 filters and 4 intermediate states) with both high spectral resolution and spatial resolution. While the other regions can be recovered in a snapshot mode (16 filters with a single intermediate state) for fast response with low spatial resolution. The simulated peak signal-to-noise ratios (PSNR) can be up to 22 dB for all modes, indicating the potential of the present method for more complex scenarios, from microscope imaging to dynamic scenes, enabling user-defined spatial and spectral resolution in regions of interest.

In conclusion, we have demonstrated a miniaturized hyperspectral imager using a reconfigurable filter array based on the VO_2_ material. Spectral reconstructed accuracy of 2.1 nm and resolution of 10 nm are proved in the experiment with a sampling rate of 4.5 Hz. Furthermore, spectral imaging is also verified with high color fidelity. For spectral imaging by filter arrays, the spatial resolution of the images is mainly determined by the sensor pixels occupied by the filter unit (i.e., the total number of filters in a unit cell). In this article, as we only use four filters in a unit, the spatial resolution of the image degrades only by a factor of 4 (in area) as compared to the original spatial resolution, and this is a huge improvement compared with a conventional filter array (where the spatial resolution can degrade by a factor of 25).^25^ Such a reconfigurable filter array combines the advantage of geometric engineering and active materials to obtain a good balance in spatial resolution and acquisition time. A comparison between different approaches can be found in Supporting Information, Section 12. The lower phase change temperature of VO_2_ makes it possible for integration and miniaturization. The present method can be easily expanded to other material platforms like chalcogenide phase change materials (PCMs)^26,27^ and the total number of filters or intermediate states can be adaptively chosen depending on different scenarios. Considering the ease of fabrication, this tunable filter array provides a possible solution for spectral imaging with both high spectral and spatial resolutions, which can be extensively employed in applications like healthcare, quality control, and environment monitoring.

## Methods

### Design and Fabrication

SiO_2_/Si substrate was fabricated by PECVD from a bare silicon substrate. The whole device fabrication process was as follows: First, the ITO heater patterns were fabricated by UV photolithography. Followed by the sputtering and lift-off process for the ITO heater. After that, we fabricated the Cr/Au electrode/wire patterns with the same method. Then the lossy cavities were fabricated by UV photolithography, followed by depositing a thin SiO_2_ layer by PECVD. Then Cr/Ag layers were sputtered sequentially. Then UV photolithography and sputtering were conducted several times to obtain Vanadium with different thicknesses. Finally, VO_2_ layers were formed by thermal annealing at 400 °C for 30 min. The VO_2_ material was confirmed by X-ray photoelectron spectroscopy and the Raman spectrum (Supporting Information, Section 1). After fabrication, a wire-bonding process was conducted to apply signals on the chip.

### Measurement and Characterization

The optical images were captured by an Olympus BX53 M microscope. To measure the reflectance of the samples, a microscope mounted with a fiber and a spectrometer (Ocean Insight, QE Pro) was used. The durability was tested by applying an AC voltage signal above 10000 cycles while monitoring the reflectance power with a photodetector (Thorlabs, PDA100A-EC) connected with an oscilloscope (RIGOL, DS1202). The response time was measured by recording the reflective intensity of the chip with a camera (ZWO, ASI432MM) while driving the chip with a pulsed signal.

### Numerical Simulation and Calculation

The optical response was simulated by ANSYS FDTD Solutions. In the simulation, the refractive indices of VO_2_ were adopted as the measured ones. Other materials were adopted from the built-in library. The reflective colors and color differences were obtained by homemade MATLAB scripts. All spectra were reconstructed by the CVX MATLAB toolbox.^28^

## Data Availability

The data that support the findings of this study are available from the corresponding author upon reasonable request.

## Supporting Information Available

The characterization of the VO_2_ material; The hysteresis effect of the VO_2_; The color gamut of the device; The response of the microheaters with different sizes; The angle-dependent response of the system; The reproducibility of the device; The calibration, measurement, and reconstruction of the spectrum; Different reconstruction performances for broadband and narrowband signals; Design rules for the optimal filters; Spatial resolution and integration; The simulation for adaptive reconstruction; Comparison of different spectral/imaging approaches (PDF)
