# Supplementary material for: Miniaturized Hyperspectral Imager Utilizing a Reconfigurable Filter Array for Both High Spatial and Spectral Resolutions
Source: Nano Lett. 2024 Aug 30;24(36):11156–62. doi: 10.1021/acs.nanolett.4c01075 (PMC11403764; doi:10.1021/acs.nanolett.4c01075)
Supplement: Supplementary file 1 — nl4c01075_si_001.pdf [file nl4c01075_si_001.pdf]

Miniaturized hyperspectral imager utilizing a reconfigurable filter array for both high spatial and spectral resolutions

Tingbiao Guo[1,2,#], Zijian Lin[1,#], Zhi Zhang[1,#], Xiao Chen[1], Yuan Zhang[1], Zhipeng Hu[1], Ruili Zhang[2] and Sailing He[2,3,4,*]

1. Centre for Optical and Electromagnetic Research, College of Optical Science and Engineering, National Engineering Research Center for Optical Instruments, Zhejiang University, Hangzhou, 310058, China

2. Taizhou Institute of Medicine, Health and New Drug Clinical Research, Taizhou Hospital, Zhejiang University, Taizhou, 318000, People's Republic of China

3. National Engineering Research Center for Optical Instruments, Zhejiang University, Hangzhou, 310058, China

4. Department of Electromagnetic Engineering, School of Electrical Engineering, KTH Royal Institute of Technology, Stockholm, SE-100 44, Sweden

[#]These authors contributed equally to this work: Tingbiao Guo, Zijian Lin, Zhi Zhang

[*]Corresponding author: sailing@kth.se

Contents

1. The characterization of the VO_2 material 2

2. The hysteresis effect of the VO_2 2

3. The color gamut of the device 2

4. The response of the microheaters with different sizes 4

5. The reproducibility of the device 6

6. The angle-dependent response of the system 7

7. The calibration, measurement and reconstruction of the spectrum 8

8. Different reconstruction performance for broadband and narrowband signals 9

9. Design rules for the optimal filters 10

10. Spatial resolution and integration 11

11. The simulation for adaptive reconstruction 12

12. Comparison of different spectral/imaging approaches. 12

1. The characterization of the VO_2 material

Figure S1a shows the refractive index of the VO_2 measured by ellipsometry (Horiba, UVISEL). Figures S1b and S1c are Raman and XRD spectra.

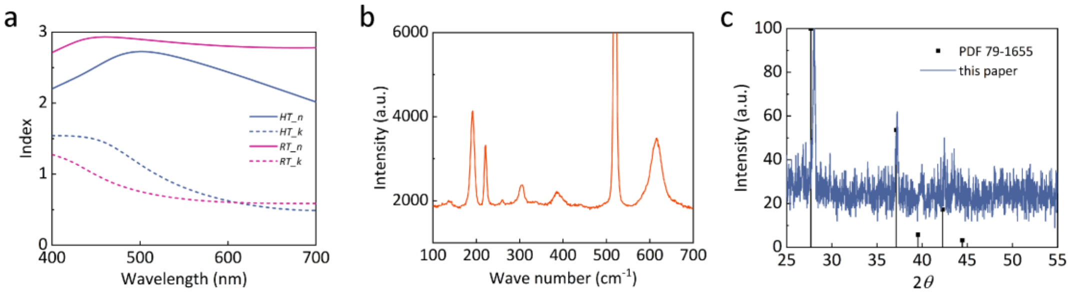

Figure S1. a.The refractive indices of the VO_2 at low (room temperature) and high (80°C) temperatures. Orange lines for Low temperature and Blue lines for High temperature. Solid lines for the real part and dashed lines for the imaginary part. b. The Raman spectrum. c. The XRD spectrum and the typical VO_2 spectrum (PDF # 79-1655).

2. The hysteresis effect of the VO_2

Figure S2 shows the hysteresis effect of our filter.

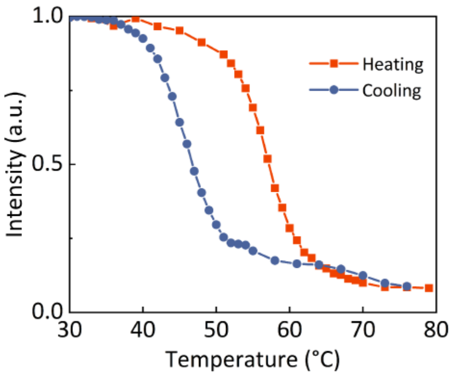

Figure S2. The hysteresis effect of the VO_2-based filter.

3. The color gamut of the device

The present Ag-VO_2 filters cover a relatively narrow color gamut. This narrow gamut comes from the *subtractive color* nature of the filters, as each filter shows an absorption peak at a relatively narrow band. In this way, the CMY color model is more commonly used rather than

a RGB model. For example, to obtain a pure R color, a broadband absorption covering both blue and green bands is needed for the filter. This can be done by other structure configurations than simple Ag-VO_2 type, like metal-insulator-metal cavity (MIM)[1] and metal-insulator-semiconductor-insulator-metal cavity (MISIM)[2]. As an example, the MISI (here the top metal layer is removed) type with Ag-SiO_2-VO_2-SiO_2 layer is simulated below. In the simulation, the thicknesses of Ag and VO_2 layers are set as 100 and 10 nm respectively. The total thickness of SiO_2 is varied from 300 to 500 nm. This configuration shows a quite large color gamut around 64.8% of the sRGB space.

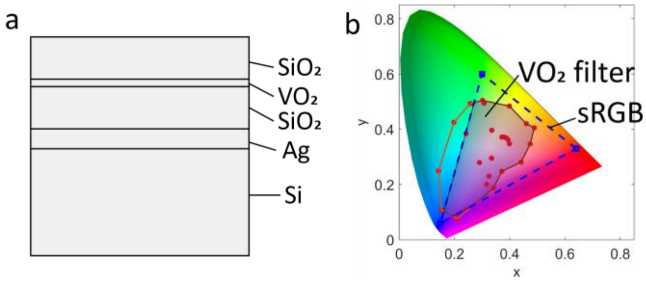

Figure S3. a. The configuration of the Ag-SiO_2-VO_2-SiO_2 filter for a large color gamut. b. The color gamut of the proposed filter in the CIE 1931 space.

To illustrate the influence of the substrate on color appearance, we conducted more simulations. As shown in the Figure below, we use platinum, aluminum, gold, and silver as substrates in the simulation. The corresponding color gamut is 7.2%, 12.1%, 23.5%, and 17.4% of the sRGB space respectively. In addition, we simulate the case of adding a layer of 50 nm SiO_2 above the VO_2 with the silver substrate, accounting for 25.6% of the sRGB space, and expanding the color of the red region. The combination of various thicknesses of VO_2 and SiO_2 layers on the Ag substrate at the same time can achieve a relatively larger color gamut, accounting for 29.8% of the sRGB space.

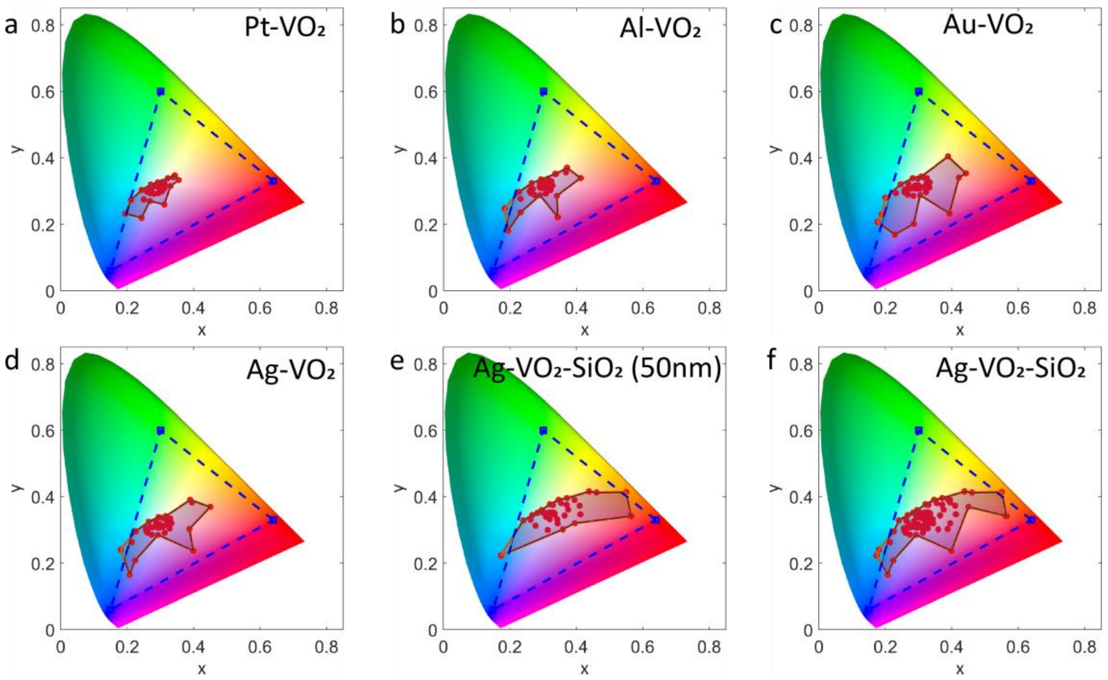

Figure S4. a-d. The color gamut of different configurations for the VO_2-based filters with different substrates. e. The color gamut of the filter with Ag-VO_2-SiO_2 (50 nm) configuration. f. The color gamut for various VO_2 and SiO_2 thicknesses with the Ag-VO_2-SiO_2 configuration.

4. The response of the microheaters with different sizes

To explore the response time of the microheater, we conducted an electrothermal analysis of the device by creating a 3D finite element model in COMSOL Multiphysics. Considering the practical situation, we set the device on a 500 μm quartz glass substrate. In our simulations, the Current Module was used to calculate the voltage distribution in the device, while the Heat Transfer in Solids Module was used to predict the temperature distribution. The heat transfer function is shown as follows. The two modules are coupled through a Multiphysics field called Joule Heating. The heating electrode we used in this paper is ITO with a conductivity of 1.1×10^5 S/m measured by the four-probe method (at room temperature). This parameter mainly affects the thermal distribution. The governing transient heat transfer equation is described by[3]:

$$\rho C_p \frac{\partial T}{\partial t} = \nabla \cdot (k \nabla T) + Q$$

where ρ is the density, C_p is the specific heat capacity, k is the thermal conductivity, T is the temperature related to time and space, and Q is the Joule heat source. The thermal parameters

used in the simulation are listed in Table S1. Except for the parameters of ITO and VO_2, the rest are using the parameters in the COMSOL material library.

Table S1. Basic material properties in thermal simulation.

Material	C_p (J/(kg·K))	k (W/(m·K))	ρ (kg/m^3)
ITO	1290	11	7090
Au	129	317	19300
SiO_2	709	1.38	2203
VO_2	205	6.46	4339

For all external boundaries, we set open thermal boundary conditions except for the quartz glass substrate and top surfaces where a heat flux condition with an ambient temperature of T_0 = 293 K and the convective heat transfer coefficient h of 10 W/(m^2·K) are considered.

In Figure S5, we show the simulated results for ITO heaters with different widths. It is evident that a smaller ITO heater can induce a faster response time. As VO_2 has many stable intermediate states, to improve the recording speed of the device, we can set different signals to obtain each intermediate state one by one with various input voltages rather than a constant voltage during the whole heating process (used in this paper). When the switching speed of the device reaches 1 ms, we only need 41×1 ms=41 ms to access 41 intermediate states, comparable to the video-rate speed (~50 ms). Furthermore, in the main text, we used all intermediate states for spectral reconstruction. We note that 20 intermediate states are enough for precise reconstruction. Thus, a faster response time was expected beyond the video rate. The reconstruction results with different intermediate states are shown in Figure S6 below for comparison.

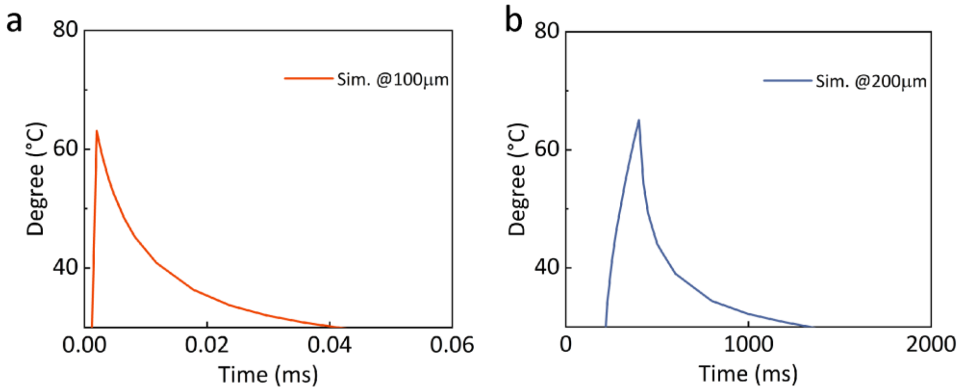

Figure S5. The simulated response of a filter with various ITO heater sizes. a: width=100 μm b: width=200 μm.

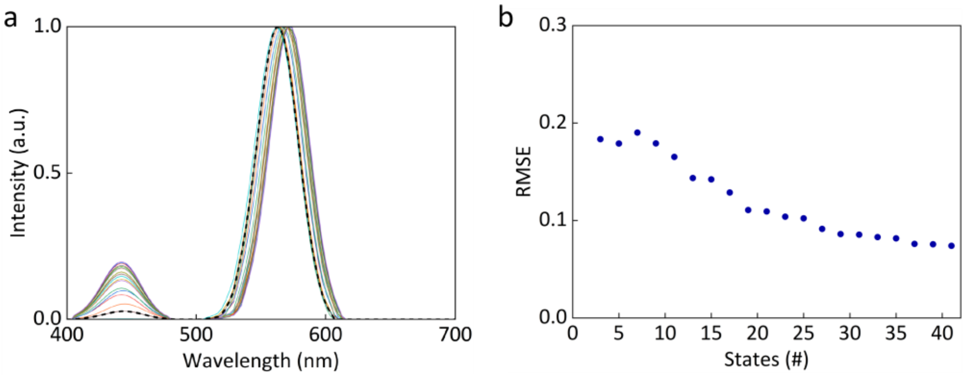

Figure S6. a. The reconstructed spectra with different intermediate states (dashed line is the ground truth) b. The RMSE of the reconstruction with different intermediate state numbers.

5. The reproducibility of the device

We measured the reflectance spectra of one filter at 30°C, 52°C, 62°C and 80°C for ten cycles in situ. The measured spectra are shown below with good repeatability.

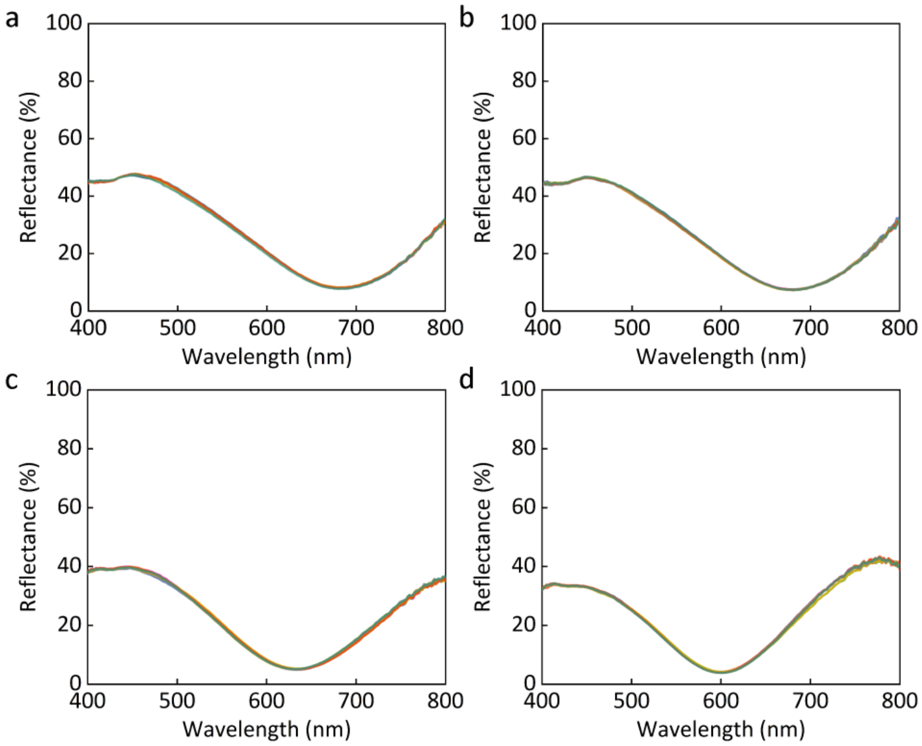

Figure S7. a-d. The reflective spectra of our filter measured by ten times at different temperatures.

Moreover, the reproducibility of the dynamic responses during the heating process was also explored. We measured the dynamic reflection intensity of one filter by heating the whole chip three times. As shown in the Figure below, the filter shows a dynamic response with good

repeatability. This makes it possible to use the transition states during the heating process for the spectrum reconstruction.

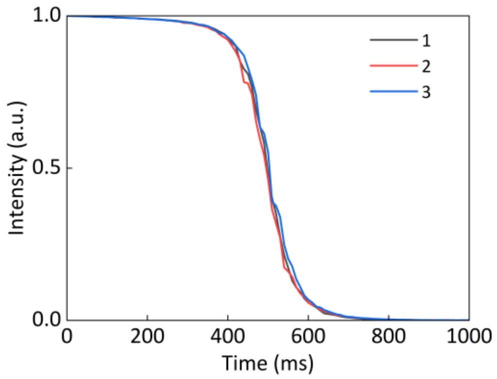

Figure S8. The dynamic reflective intensity of our filter measured during the heating process three times.

6. The angle-dependent response of the system

The angle-dependent response of our filter was simulated with FDTD. In the simulation, light sources with incident angles of 5, 10, 15, 20 degrees were used. The thickness of VO_2 was set as 50 nm. The result in Figure S9 shows our filter is intensive to the incident angle within a range of (-20, +20) degrees. In fact, many previous works in the literature have shown that a lossy material is beneficial for a large incident-angle range as it will induce a nontrivial phase shift and lead to a thinner cavity[4]. Furthermore, we have measured the reflective responses by objectives with various Numerical Aperture (NA) (here we use NA to represent incident angles). In the measurement, a spectrometer with a pigtail fiber pigtail (NA=0.38, angle=22°) was mounted onto the microscope. We used objectives with NA=0.1, 0.3, 0.45 and 0.5 to measure the reflectance of our filter. The measured results are shown in the Figure below. As can be seen, no evident wavelength shift is found with different objectives, validating the simulation results. As we used an objective of NA=0.3 (~17.5°) for all experiments in the main text, the angle-dependent effect can be ignored.

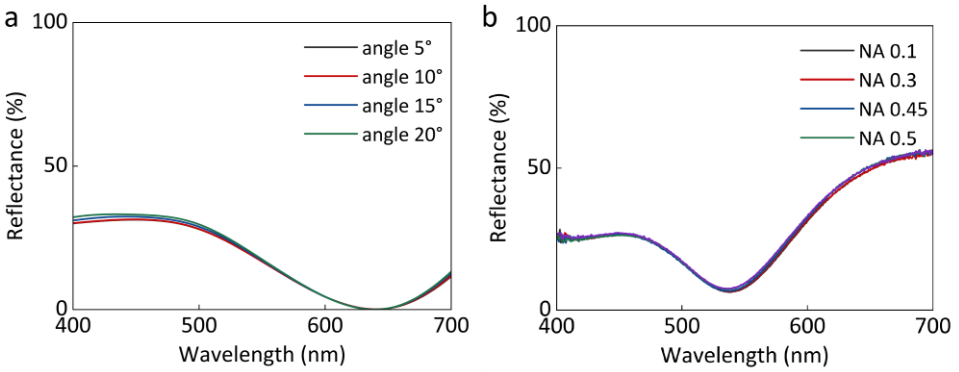

Figure S9. a. The simulated reflectance of our filter under different incident angles. b. The measured reflectance of our filter with different objectives.

7. The calibration, measurement and reconstruction of the spectrum

Calibration

The reflection spectra of TRFA were calibrated on an Olympus microscope (BX53M). We obtained monochromatic light sources of various colors by placing a series of narrowband filters behind the white light source of the microscope. The bandpass filter had a bandwidth of ten nanometers, covering the entire visible wavelength band. TRFA was driven by a signal pulse output from a signal generator (SIGLENT, SDG1062X) and the response was recorded by a monochromatic camera (ZWO, ASI432MM). Since the chromatic aberration of the optical system can’t be ignored, the focal length of the microscope was adjusted to make the image of TRFA clearest when using different wavelengths of light for illumination, which introduced a lot of errors. Raw data obtained by the camera required image translation, scaling, and other operations based on the transmittance of the filter and the displacement generated when focusing.

Measurement

The measurement system was built as shown in Figure 4 in the main text. The object to be measured was placed on the conjugated plane. The pulse voltage with a period of 4 seconds, the duty cycle of 50%, and the amplitude of 4 V was applied on the chip. In the meantime, the camera continuously recorded the images. The camera was set as an exposure time of less than ten milliseconds and a frame rate of approximately 88 frames per second.

Reconstruction

The recovery of the measured spectra was done by MATLAB R2023a and the CVX toolbox was used to solve equation 2. We first found the position of each block and then used 3×3 pixels in the middle of each block as calibration/ measurement values to decrease the noise at the edges. The values of each block were arranged in the time dimension, and the gradient was calculated to determine the state of phase change at each time point. In this way, all pixels were aligned in time and space respectively. In order to prevent over-fitting, we used the ridge regression in the solving process and the regularization coefficient of the regularization term was 0.05. Rebuilding an image with 3363 pixels will take approximately 19 minutes (100 bands in each pixel spectrum, CPU is AMD Ryzen 5 4600H with Radeon Graphics, 3.00 GHz).

8. Different reconstruction performance for broadband and narrowband signals

For our spectral imaging system, the reconstruction accuracy is influenced not only by the filter design but also by the calibration and measurement processes. As can be seen in Figure S10 below, the intensity of the light source used in our system at 450 nm and 580 nm bands is much larger than other wavelengths. Due to this imbalanced intensity of our light source, when measuring broadband signals, wavelength bands other than 450 nm/580 nm will get a smaller signal-to-noise ratio. This low signal-to-noise ratio deteriorates the reconstruction performance for broadband signals. But for narrowband signal measurement, we can either increase the intensity of the light source or increase the integral time to increase the signal-to-noise ratio. Thus, the reconstruction performance can be improved for narrowband signals.

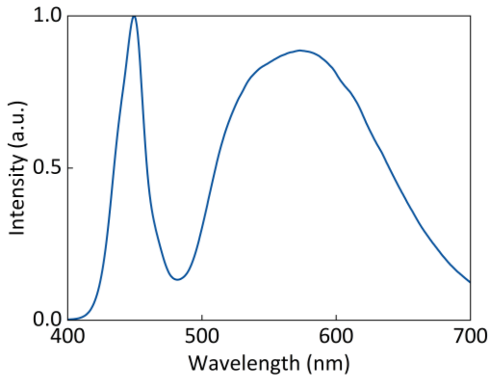

Figure S10. The relative spectral energy distribution of the light source used in our system.

To demonstrate its ability for broadband spectra, we show more reconstruction results for broadband spectra in Figure S11. The reconstruction RMSE is 0.079 for broadband spectrum. The result is close to the results in the main text (RMSE=0.123).

For further work, a more accurate calibration and measurement process can improve the reconstruction performance. For example, a more balanced flat-band light source may help this. Moreover, the relatively worse accuracy for broadband signals may be improved with more delicate regularization terms as only the L1 regularization term is used in our paper, which is more suitable for sparse signal recovery.

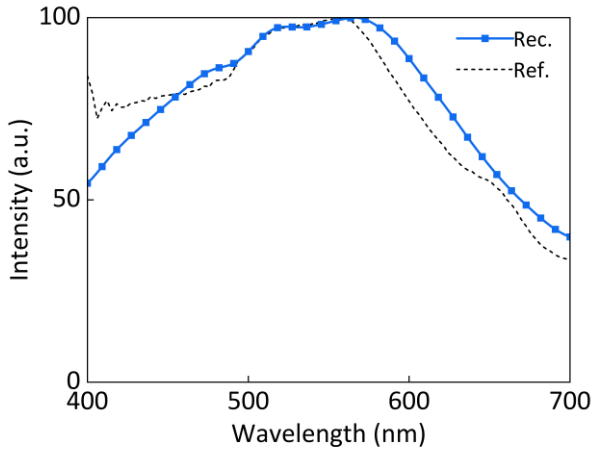

Figure S11. a. The reconstruction performance for the broadband spectrum.

9. Design rules for the optimal filters

To improve the reconstruction performance, one possible way is to use an optimization method for the filter design. Below we show the reconstruction performance of different filter groups designed by different Figures of Merit (FoMs) by the particle swarm optimization (PSO). In the simulation, the reflection of 100 filters with VO_2 thickness varying from 20-200 nm is first calculated as a spectrum library. Each filter is assumed to have 3 intermediate states. Different FoMs include: 1) minimizing the average coefficients for the chosen 4 filters; 2) minimizing maximum coefficients for the chosen 4 filters; 3) randomly choosing 4 filters were used for choosing 4 filter combinations. For the spectrum reconstruction simulation, 31 Gaussian-shape spectra with sigma=50 nm were used as input signals. The spectra were reconstructed based on the equation below:

$$I = \arg\min_{I}(||HI - P||_2 + \lambda_1||I||_1 + \lambda_2||I||_2)$$

where λ_1 and λ_2 are regulation terms and were chosen by minimum the mean RMSE of between reconstructed signals and the ground truth. ***I*** means the reconstructed spectrum. ***H*** is the measurement matrix. Here the chosen 4 filters with 3 intermediate states are using to build the ***H***. ***P*** is the detected power of the detector.

The reconstruction performance is evaluated by the mean RMSE as shown below. It is evident that minimizing the average coefficients for the chosen 4 filters is a better choice for the spectrum reconstruction.

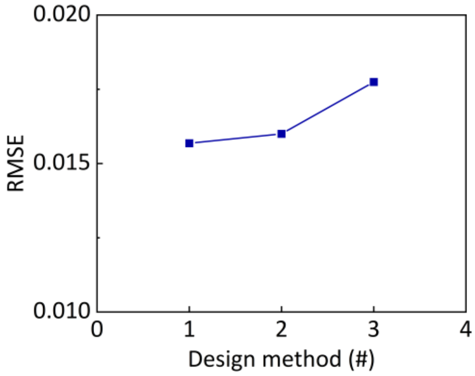

Figure S12. The reconstruction performance with different design methods.

10. Spatial resolution and integration

For simplicity, in our paper, we magnify the temporally reconfigurable filters 10 times by an objective and re-image it on a colorful object. Due to the limited number of filter pixels per area on the object, the spatial resolution for spectral imaging is largely decreased. In particular, at the edge of some areas, a filter has been divided into 2 parts by the object, thus causing some errors in the reconstruction of these pixels. In addition, fabrication errors (uniformity and defects) and calibration errors also lead to worse accuracy.

As our work utilizes fewer filters (only 4), it can lead to better potential spatial resolution comparable to the common color camera (Bayer filter type). For future work, a high-quality transmissive reconfigurable filter pixel-wise fabricated on a CCD/CMOS sensor may help to improve the spatial resolution and make the device more compact. For example, one can insert a thin VO_2 layer in a FP cavity for transmissive design[5].

11. The simulation for adaptive reconstruction

In the simulation shown in the main text Figure 5, 100 filters with different SiO_2 and VO_2 thicknesses (Figure S13) were simulated with ANSYS FDTD Solution software. The thickness of SiO_2 ranges from 20 nm to 200 nm and the thickness of VO_2 ranges from 20 nm to 200 nm. Five intermediate states of the VO_2 material were simulated with refractive index $n = fn_\mathrm{l} + (1-f)n_\mathrm{h}$, where n_l is the refractive index of the VO_2 in the insulating state and n_h is the refractive index of VO_2 in the metallic state. *F* means the volume ratio of the phase transition part. We used *f* from 0 to 1 to represent different intermediate states. 16 filters with minimum average correlation coefficients are chosen by the PSO algorithm.

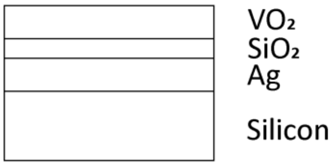

Figure S13. The schematic of the simulated filter.

To calculate the RMSE with different filter combinations, the spectra are reconstructed based on the equation below:

$$\boldsymbol{I} = \arg\min_{\boldsymbol{I}}(||\boldsymbol{HI}-\boldsymbol{P}||_2 + \lambda_1||\boldsymbol{I}||_1 + \lambda_2||\boldsymbol{I}||_2)$$

λ_1 and λ_2 are chosen by minimum the mean RMSE of $\boldsymbol{I}$ by using 100 random spectra as input signals.

We used the CAVE database in the spectral image simulation. In the simulation, the green balls labeled in the ROI were chosen according to their shape and color images were rendered by homemade scripts. And λ_1 and λ_2 were chosen by minimum the mean RMSE of $\boldsymbol{I}$ by using 100 points randomly picked from the image. In the ROIs, the spectra were reconstructed by using 4 filters with 5 intermediate states while other parts were reconstructed by using 16 filters with 1 state.

12. Comparison of different spectral/imaging approaches.

Compared with the spatially modulated filters, the proposed filter has a higher spatial resolution due to fewer filter numbers in a unit cell, comparable to the RGB camera. Due to the simple

fabrication process and configuration, it shows low cost and simple system complexity, this is especially important for portable devices. Moreover, the response time of the proposed filter can reach to video rate with precise thermal management.

Table S2. Comparison of different spectral/imaging approaches.

**Year**	**Structure**	**Spatial resolution (filter numbers/unit)**	**Spectral resolution (nm)**	**Cost**	**Complexity**	**Speed**	**Ref.**
**Spatial modulation**							
2000	FP cavity	16	16	Low	Low	**Fast**	**[6]**
2019	Photonic crystal	36	1.4	Medium	Medium	**Fast**	**[7]**
2022	Metasurface	25	0.8	Medium	Medium	**Fast**	**[8]**
2023	FP cavity	64	10	Low	Low	**Fast**	**[9]**
2023	Dye-doped photoresist	16	2.65	Low	Medium	**Fast**	**[10]**
**Temporal modulation**							
2002	Liquid crystal	1	<10	High	High	Medium	**[11]**
2002	Acousto-optical material	1	<10	High	High	**Fast**	**[12]**
2020	Phase change material	1	74	**Low**	High	Slow	**[13]**
2022	Perovskite	1	5	**Low**	Medium	Slow	**[1514]**
2022	2D material	1	<3	**Low**	High	Slow	**[15]**
**Temporally reconfigurable modulation**							
2024	Electrochromic material	12	3	**Low**	Medium	Slow	**[16]**
**Ours**	VO_2	4	10	**Low**	**Low**	Medium	

References

1. Yang, Zhengmei, et al. "Reflective color filters and monolithic color printing based on asymmetric Fabry–Perot cavities using nickel as a broadband absorber." Adv. Opt. Mater. 4.8 (2016): 1196-1202.
2. Kim, Jaeyong, et al. "Generation of reflection colors from metal–insulator–metal cavity structure enabled by thickness-dependent refractive indices of metal thin film." ACS Photonics 6.9 (2019): 2342-2349.
3. Chen, Xi, et al. "Nanosecond photothermal effects in plasmonic nanostructures." ACS Nano 6.3 (2012): 2550-2557.
4. Kats, Mikhail A., et al. "Nanometre optical coatings based on strong interference effects in highly absorbing media." Nature Materials 12.1 (2013): 20-24.
5. Xiang, Jiewei, et al. "Ultrabroadband, high color purity multispectral color filter arrays." ACS Photonics (2023).
6. Correia, J. H., et al. "Single-chip CMOS optical microspectrometer." Sensors and Actuators A: Physical 82.1-3 (2000): 191-197.
7. Wang, Zhu, et al. "Single-shot on-chip spectral sensors based on photonic crystal slabs." Nature Communications 10.1 (2019): 1020.
8. Xiong, Jian, et al. "Dynamic brain spectrum acquired by a real-time ultraspectral imaging chip with reconfigurable metasurfaces." Optica 9.5 (2022): 461-468.
9. Yako, Motoki, et al. "Video-rate hyperspectral camera based on a CMOS-compatible random array of Fabry–Pérot filters." Nature Photonics 17.3 (2023): 218-223.
10. Bian, Liheng, et al. "A low-cost integrated hyperspectral imaging sensor with full temporal and spatial resolution at VIS-NIR wide range." arXiv preprint arXiv:2306.11583 (2023).
11. Hardeberg, Jon Y., Francis Schmitt, and Hans Brettel. "Multispectral color image capture using a liquid crystal tunable filter." Optical engineering 41.10 (2002): 2532-2548.
12. Gupta, Neelam, Rachid Dahmani, and Steve Choy. "Acousto-optic tunable filter based visible-to near-infrared spectropolarimetric imager." Optical Engineering 41.5 (2002): 1033-1038.
13. Julian, Matthew N., et al. "Reversible optical tuning of GeSbTe phase-change metasurface spectral filters for mid-wave infrared imaging." Optica 7.7 (2020): 746-754.
14. Guo, Linqi, et al. "A single-dot perovskite spectrometer." Advanced Materials 34.33 (2022):

2200221.

15. Yoon, Hoon Hahn, et al. "Miniaturized spectrometers with a tunable van der Waals junction." Science 378.6617 (2022): 296-299.

16. Tian, Menghan, et al. "Miniaturized on-chip spectrometer enabled by electrochromic modulation." arXiv preprint arXiv:2402.18935 (2024).
